# Challenges and opportunities in establishing a collaborative multisite observational study of chronic diseases and lifestyle factors among adults in Puerto Rico

**DOI:** 10.1186/s12889-017-4035-z

**Published:** 2017-01-31

**Authors:** Josiemer Mattei, José F. Rodríguez-Orengo, Martha Tamez, Francheska Corujo, Aida Claudio, Héctor Villanueva, Hannia Campos, Walter C. Willett, Katherine L. Tucker, Carlos F. Ríos-Bedoya

**Affiliations:** 1 Department of Nutrition, Harvard TH Chan School of Public Health, 665 Huntington Ave, Bldg 2, Boston, MA 02115 USA; 2 Fundación de Investigación de Puerto Rico, San Juan, PR USA; 3 Department of Biochemistry, School of Medicine, Medical Sciences Campus, University of Puerto Rico, San Juan, PR USA; 4 HealthProMed, Santurce, PR USA; 5 Centro de Investigación e Innovación en Nutrición Translacional y Salud, Universidad Hispanoamericana, San José, Costa Rica; 6 Department of Epidemiology, Harvard TH Chan School of Public Health, Boston, MA USA; 7 Department of Biomedical and Nutritional Sciences, University of Massachusetts, Lowell, MA USA; 8 Hurley Medical Center, Department of Internal Medicine, Flint, MI USA

**Keywords:** Puerto Rico, Observational studies, Collaborative work, Partnerships, Health disparities, Chronic diseases, Lifestyle behaviors, Population health, Community health, Process evaluation

## Abstract

**Background:**

Prevalence of chronic diseases and unhealthy lifestyle behaviors among the adult population of Puerto Rico (PR) is high; however, few epidemiological studies have been conducted to address these. We aimed to document the methods and operation of establishing a multisite cross-sectional study of chronic diseases and risk factors in PR, in partnership with academic, community, clinical, and research institutions.

**Methods:**

The Puerto Rico Assessment of Diet, Lifestyle and Diseases (PRADLAD) documented lifestyle and health characteristics of adults living in PR, with the goal of informing future epidemiological and intervention projects, as well as public health, policy, and clinical efforts to help improve the population’s health. The study was conducted in three primary care clinics in the San Juan, PR metropolitan area. Eligible volunteers were 30–75y, living in PR for at least 10 months of the previous year, and able to answer interviewer-administered questionnaires without assistance. Questions were recorded electronically by trained interviewers, and included socio-demographic characteristics, lifestyle behaviors, self-reported medically-diagnosed diseases, and psychosocial factors. Waist and hip circumferences were measured following standardized protocols. A subset of participants answered a validated food frequency questionnaire, a legumes questionnaire, and had medical record data abstracted. Process and outcome evaluation indicators were assessed.

**Results:**

The study screened 403 participants in 5 months. Of these, 396 (98%) were eligible and 380 (94%) had reliable and complete information. A subset of 242 participants had valid dietary data, and 236 had medical record data. The mean time to complete an interview was 1.5 h. Participants were generally cooperative and research collaborators were fully engaged. Having multiple sites helped enhance recruitment and sociodemographic representation. Diagnosed conditions were prevalent across sites. Challenges in data monitoring, interviewer training, and scheduling were identified and corrected, and should be addressed in future studies.

**Conclusions:**

Epidemiological studies in PR can be successfully implemented in partnership with multiple institutions. Effective recruitment and implementation requires concerted planning and continued involvement from partners, frequent quality control, brief interviews, reasonable incentives, and thorough training/re-training of culturally-sensitive interviewers. Further studies are feasible and needed to help address highly prevalent chronic conditions in PR.

## Background

Recent reports from Puerto Rico (PR), a United States (US) territory, highlight an adverse health profile among the island’s general population. The top leading causes of death in PR are all chronic diseases: heart diseases, cancer, diabetes, and Alzheimer’s disease, and overall death rates are surpassing birth rates [1]. Among the most prevalent conditions were arthritis (20%), depression (17%), and diabetes (14%) [2]. Even though it has been estimated that more than 90% of type 2 diabetes, 80% of heart disease, 70% of stroke, and 70% of colon cancer may be prevented by not smoking, maintaining a healthy weight, engaging in moderate physical activity, and consuming a healthy diet with moderate alcohol intake [3], risky lifestyle behaviors are widespread in PR. In 2011, 2 out of 3 individuals were classified with overweight or obesity, and 15% of the population smoked [2], with increasing trends in the past few years [1]. Sociodemographic disparities were reported for the same time period, with low median household income ($18,660) and high unemployment (16%), despite high percentages of college (or above) education (45%) [1]. With over 3.5 million inhabitants in the densely-populated island of PR, tending to the health of the population must be a priority.

Public health and clinical initiatives have the potential to reduce morbidity and mortality in chronic diseases and risk factors. The most successful prevention initiatives are based on scientific evidence [4, 5]. For chronic diseases and their risk factors, much of this evidence has come from epidemiological studies of observational and intervention design [5, 6]. Observational studies are particularly appropriate to provide useful information when trials and interventions are unethical or unfeasible, and to generate hypotheses and preliminary evidence to better design future studies [7]. For example, the Framingham Osteoporosis Study has helped identify multiple nutrients and foods that alter bone health [8], some of which are now being applied in dietary trials. Similarly, even though major risk factors for cardiovascular disease have been well-known for decades, the Hispanic Community Health Study/Study on Latinos (HCHS/SOL) recently documented large variations in dietary intake patterns [9, 10] and cardiometabolic risk among diverse US Hispanic/Latino background groups, highlighting a need to focus in preventive health behaviors by specific ethnic group [11, 12]. This last example underscores the importance of epidemiological studies in revealing new information that can better inform multi-level public health efforts tailored to the target population.

Despite the need to understand chronic disease contributors in PR, there is a dearth of concerted efforts in the island to study the determinants and dynamics that drive chronic diseases as a way to identify potential ways to prevent them. The most comprehensive epidemiological study conducted in the island was the Puerto Rico Heart Health Program in the 1970s, which was designed to investigate morbidity and mortality from coronary heart disease in Puerto Rican men [13]. Important results on dietary risk factors [14–16], sociodemographic and lifestyle risk factors [17–21], and biological markers [22, 23] were documented for cardiovascular disease, diabetes, and other chronic diseases. However, no large-scale comprehensive studies have been conducted on the island in the 45 years since then, despite the documented rapid increases in chronic diseases and unhealthy lifestyles. Subsequent studies have collected data on chronic conditions or risk factors, but have been limited by scope or target sub-population. For example, the Puerto Rican Elderly: Health Conditions longitudinal, island-wide study investigated social issues affecting the elderly (≥60y) from 2002 to 2007 [24]. The San Juan Obesity Longitudinal Study prospectively followed San Juan residents aged 40–65y with overweight or obesity in 2005 [25]. A cross-sectional study of Puerto Ricans aged 21–79y residing in the San Juan metropolitan area successfully collected information in 2005 on prevalence of chronic diseases and risk factors using questionnaires and physical and laboratory measures [26, 27]. Few of these studies documented the process and lessons learned while establishing the cohorts, making it difficult for other investigators to estimate the resources, time, and efforts needed to conduct similar studies.

Further rigorous efforts to collect valid data on multi-level contributors to chronic disease are needed to address the considerable health disparities in PR. Moreover, documenting the operational process and methodology can help prevent or correct problems and improve the success of future studies. Initiatives carried out through partnerships are of particular value to help share interdisciplinary research ideas, advocacy, education and training, expertise, capacity-building, resources, and funding [4, 28]. Thus, the aim of this report is to document the methodology, process and outcome evaluation, and lessons learned by conducting the Puerto Rico Assessment of Diet, Lifestyle, and Diseases (PRADLAD), a collaborative multisite cross-sectional study aiming to assess lifestyle factors and prevalence of chronic diseases among adults in PR. PRADLAD has the goal of informing future epidemiological and intervention projects, as well as public health, policy, and clinical efforts, to help improve the population’s health.

## Methods

### Study design and population

PRADLAD was designed as a cross-sectional survey of a convenience sample of adults living in PR. The study was advertised at the main entrances and waiting rooms of three partner clinics in the San Juan metropolitan area: Fundación de Investigación de PR (FDI; a research center and medical care facility), Centro Multidisciplinario de Medicina de la Asociación de Maestro de PR – CDT Asociación de Maestro de Hato Rey (a clinic within a city hospital, Hospital del Maestro), and Health Pro Med (a nonprofit federally-funded primary community health care center). Recruitment was conducted in the primary care sites of the clinics, and not in specialized care units. Study personnel distributed recruitment flyers to individuals in the waiting room of the primary care clinics. FDI was the study’s main partner on the island, and they had previous successful research and clinical collaborations with the two other clinics. These two partner clinics were selected because they were committed to the project and had the necessary resources (i.e.: private interview rooms, internet access, sharable medical records), and to increase representation of sociodemographic characteristics of participants. The study aimed to recruit 450 participants and expected a conservative 20% loss due to lack of eligibility and incomplete or poor data, based on a previous study of Puerto Ricans from Boston [29], for a final sample size of 360 participants.

Participants were patients waiting for a medical appointment, or persons accompanying a patient, or visitors at one of the clinics. Interested individuals talked with an identified study research assistant, who provided details about the study and a screening questionnaire to confirm eligibility. To be eligible, an individual had to be living on PR at the time of the study and for at least 10 months of the previous year, be aged 30–75y, and be able to answer questions without assistance (i.e.: no major speech or neurological impairment). The study was conducted between July and November, 2015. All participants provided written informed consent. The Institutional Review Board (IRB) at Harvard T.H. Chan School of Public Health, Ponce Health Sciences University in PR, University of Massachusetts, and Northeastern University, approved the study.

### Data collection

Questionnaires were administered by trained, Spanish-speaking research assistants in a private room at the clinics while the participant waited for their medical appointment (if a patient at the clinic). If the interview was not completed at that time, the participant had the option to complete the interview after the appointment or by telephone (for which contact information was obtained), or to terminate the interview (which they were notified they could do at any time). Any participant who did not complete the interview during the initial contact was re-contacted up to 3 times by phone within the subsequent 14 days to finish the interview.

Study data were collected and managed using the electronic data capture tool ‘Research Electronic Data Capture’ (REDCap) hosted at Harvard T.H. Chan School of Public Health. REDCap is a secure, web-based application designed to support data capture for research studies, providing 1) an intuitive interface for validated data entry; 2) audit trails for tracking data manipulation and export procedures; 3) automated export procedures for seamless download of data to statistical packages; and 4) procedures for importing data from external sources [30]. In addition, interviewers had hardcopies of the questionnaires as backup.

The interview consisted of 3 questionnaires: 1) a main questionnaire that asked questions on demographic and socioeconomic characteristics, medical history, lifestyle behaviors, and psychosocial measures, 2) a food frequency questionnaire (FFQ) that included questions on the frequency and quantity of intake of a list of foods and beverages, and 3) an optional, supplemental legumes questionnaire that asked about perceptions towards bean intake. Eligible participants were asked for their consent to obtain a copy of their medical records, from which we extracted information on anthropometry, blood pressure, medical diagnoses, medication use, and recent laboratory results. They were also asked to consent to being contacted for future research opportunities or additional questions, and to having their waist and hip circumference measured. Participants received a one-time $20 gift card incentive for completing the main questionnaire and FFQ. Participants who opted to answer the legumes questionnaire received an additional $5. Additionally, we provide each participant with a bag of healthy snacks and water. The questionnaire was written in Spanish using Puerto Rican vernacular, and was pre-piloted for clarity among Puerto Rican adults before implementation. Participants were informed that completing all sections would take approximately 1.5 h.

All interviewers were thoroughly trained by experienced staff to administer the consent form and questionnaires, and to perform anthropometric measurements. Each interviewer was required to practice interviews before collecting data. Open-ended questions at the end of each section allowed the interviewer to provide more information about the participant and feedback on the interview process.

#### General background characteristics

Participants provided information on demographic and socioeconomic characteristics including household composition, educational attainment, marital status, work history, household income, food security and food assistance, and use of communications technology. Questionnaires used to collect these data were based on the Boston Puerto Rican Health Study (BPRHS) [29] and the National Health and Nutrition Examination Survey (NHANES) [31], with some modifications by the research team for clarity within this population.

#### Health and health behaviors

Participants were asked whether they had ever received a diagnosis by a doctor or health professional of a specific list of chronic conditions [29]. If a participant responded affirmatively to having a disease, we obtained detailed information on medications, time of diagnosis, and current status of the disease. Additionally, we asked participants with a diagnosis of major cardiometabolic conditions to report if they had received and/or followed medical advice on diet, physical activity, or medication treatments for each condition [32]. We asked female participants for information on pregnancy and menopausal status, and all participants for family history of major chronic diseases, use of health services, health insurance, and self-rated health status, as adapted from questions from BPRHS and NHANES [29, 31].

We assessed history, frequency, quantity, and type of smoking and alcohol use [29]. Physical activity was captured using a modified Paffenbarger questionnaire of the Harvard Alumni Activity Survey [33], which was effectively tested in a middle-aged Puerto Rican population [29]. A physical activity score was calculated as the sum of hours spent on typical 24-h activities (heavy, moderate, light, or sedentary activity, and sleeping) multiplied by weighing factors that parallel the rate of oxygen consumption associated with each activity [29]. Physical activity levels were defined as sedentary (score < 30), light (30 to <40), or moderate or vigorous (≥40). Questions on hours of sleep over a 24-h period and difficulty falling asleep [34] were included; these were previously asked among Puerto Ricans [35].

### Nutrient intake and dietary behaviors

A semi-quantitative FFQ was used to assess dietary intake. The FFQ has been adapted and validated for this population [36, 37]. Those with energy intakes < 600 or > 4800 kcal and/or 2 or more sections of the questionnaire left blank were excluded from dietary analyses. The FFQ were administered in REDCap [30] and the file was linked with the Minnesota Nutrient Data System (version 5.0_35) for food and nutrient analyses.

A comprehensive questionnaire was developed to assess dietary behaviors and attitudes. Questions were adapted from the Food Attitudes and Behaviors Survey of the National Cancer Institute [38, 39], questions asked in HCHS/SOL [40], and a validated dietary behaviors questionnaire for Latinos [41], with additional questions developed by the research team for further validation in this study. The Dietary Behaviors domain included questions on food shopping and cooking roles; time, frequency, and place of meals; frequency and choices when eating away from home; cooking and eating practices; self-rated dietary quality; dietary identity; drinking water choice and safety; and nutrition awareness and knowledge. The Dietary Attitudes domain included questions on reasons for general food choices; attitudes and beliefs towards healthy eating; and motivations, self-efficacy, and intentions for healthy eating.

#### Anthropometry

Self-reported weight, height, and systolic and diastolic blood pressure were queried. Participants who consented, were not pregnant at the time of the interview, and were able to have anthropometric measures, were measured for waist and hip circumferences by the trained interviewer using a Gulick measuring tape following standard protocols [31, 42]. Measures were taken with the participant relaxed, standing with feet close together, arms at the side and body weight evenly distributed, and at the end of a normal expiration. The participant was asked to remove any additional layers of clothing and lift the shirt slightly. Waist circumference was measured at the midpoint between the lower margin of the least palpable rib and the top of the iliac crest. Hip circumference was measured around the widest portion of the buttocks, with the tape parallel to the floor. If a waist line could not be detected, the participant was asked to indicate the location of his/her umbilicus at which point the measure was taken. Measurements were taken in centimeters to the nearest millimeter, as well as in inches to the nearest decimal, in duplicate. A third measurement was taken if there was more than 1 cm (0.4 in.) of difference between the first and second measurement. We used the average of the two or three measurements as the final value.

Waist circumference thresholds used to define abdominal obesity were >102 cm in men or >88 cm in women, according to US guidelines, with a second cutoff (>94 cm in men, >80 cm in women) recommended for populations of European and Sub-Sahara African heritages [42]. We calculated the waist-to-hip ratio by dividing the waist by the hip measurement; a waist-to-hip ratio of ≥0.90 in men or ≥0.85 in women was deemed as elevated [42]. Body mass index (BMI) was calculated by dividing self-reported weight in kilograms by height in meters squared. BMI was used to classify participants according to their weight status: underweight (15.0 to 18.4 kg/m^2^) [43, 44], normal weight (18.5 to 24.9 kg/m^2^), overweight (25.0 to 29.9 kg/m^2^), or obesity (≥30 kg/m^2^) [42].

#### Psychosocial scales

We used the Center for Epidemiology Studies Depression (CESD) Scale to assess depressive symptomatology, [45] based on how often a participant agreed with 20 statements about feelings within nine domains. The scale ranges from 0 to 60; a higher score corresponds to stronger depressive symptomology. The CESD has shown good reliability and validity in Hispanics, including Puerto Ricans [46–48], although less so for Puerto Rican low-income men [49]. Depressive symptomatology was defined as CESD score ≥16 [45–47]. The Perceived Stress Scale measures the degree to which people perceive their lives as stressful based on 14 statements [50]; the score ranges from 0 to 56 with higher scores indicative of higher stress. This scale has been satisfactorily evaluated in Spanish-speakers, including Puerto Ricans [51–53]. We assessed overall perceived social support with the 12-item Interpersonal Support Evaluation List (ISEL-12), which has an overall range of 0–36; greater scores relate to higher social support. The scale includes three subscales: appraisal (advice or guidance), belonging (empathy, acceptance, concern), and tangible support (help or assistance, such as material or financial aid) [54]. The ISEL-12 has shown reliability and validity among Hispanics including Puerto Ricans [55]. Participants who reported a diagnosis of diabetes were asked 5 questions derived from the Diabetes Social Support Questionnaire-Family Version [56] to assess perceived family support for diabetes management. A higher score within a 0–25 range indicates higher diabetes support.

#### Legumes questionnaire

The supplemental legumes questionnaire was part of a second study on attitudes and preferences of beans, with the long-term goal of informing a bean-promotion intervention in the island. The questionnaire was developed from one previously implemented in PR, [57] with additional questions adapted from the Food Attitudes and Behaviors Survey.

### Statistical analysis

Descriptive characteristics by clinic were assessed using SAS software version 9.4 (SAS Institute Inc; Cary, NC). Differences among clinics were tested using chi-square for categorical variables or analysis of variance for continuous variables. Statistically significant differences were considered at a two-tailed alpha <0.05.

### Process and outcome evaluation

An initial pilot was conducted the first week of the study to gauge flow of enrollment and duration of interviews, and to identify and correct issues presented in the field. The main research team held weekly or biweekly meetings to debrief the progress of the study. A form to report adverse events was developed. Recruitment logs were maintained on-site at FDI and checked by two research assistants and a research coordinator. Data quality checks were done by two investigators every 1–3 weeks: for every 25 recruited participants until the first 100, and then every 50 participants. Training or retraining sessions were conducted whenever issues were identified or when a new staff member joined the study.

Indicators for process evaluation included recruitment logs, reports of adverse events, and number of trainings/re-trainings. In addition, written open-ended comments from interviewers and participants collected in the interviews were used to gauge feedback from participants, and team meeting notes were used to identify challenges and corrective actions. Indicators for outcome evaluation included number of participants recruited, number of eligible participants, duration of the interviews, duration of the study, completion and quality of the data collected and results of main sociodemographic and health characteristics of participants by clinic.

## Results

### Process evaluation indicators

Eleven interviews were completed in the pilot week. Immediate debriefing identified that the interviews were longer than anticipated, averaging 1 h and 45 min (median: 1:33) when conducted in person, and that some anthropometric measurements were done incorrectly, despite interviewers having had two trainings and practice sessions. Team meetings also identified issues with unreliable Internet connection that interrupted the interviews. Some interviewers reported that the psychosocial questions elicited emotional reaction from a few participants. In response, we removed selected sections of the questionnaire, and retrained interviewers to measure waist and hip circumference, to properly ask the psychosocial questions, and to sympathetically handle any emotional reaction or to skip the questions or stop the interview if a participant became visibly upset.

Recruitment through subsequent weeks was steadily high, as we had 11 interviewers working full time. There was a decline in participants’ recruitment from weeks 4 to 9, corresponding to the beginning of the academic year for many of our interviewers who cut back to part-time. To boost our recruitment goals, we identified and trained 11 new interviewers who started working on week 10 and sustained an average of 23 participants per week for the remainder of the study.

Four trainings/re-trainings were needed during the course of the study. Team meetings and data quality checks identified mistakes in data entry: failing to take the anthropometric measures twice or to enter the decimal point, failing to mark any non-applicable or blank answers, not verifying that the hours for the physical activity questions added to a 24 h period, and entering the phone numbers or the time of interview in the wrong format. The data quality check team corrected most of these mistakes.

Feedback from the open-ended entries suggested that emotional reactions still occurred during the psychosocial questions (i.e.: teary-eyed, or sharing their situation and feelings with the interviewer), especially among those with diagnosed depression. However, no interviews had to be stopped because of a participant becoming upset, and we did not have any reports of adverse events. Participants commented that they found some of the questions repetitive and the interviews long, despite them averaging 1:33 h (median: 1:24) after deleting sections in the first week. Still, comments reflected a positive attitude from participants, who were generally cooperative and answered enthusiastically. Interviewers mentioned that the incentives were appealing and the snacks provided were well received, especially as they waited for their appointment.

Through the course of the study, the partner clinics remained engaged and cooperative to the study needs by providing access to private rooms, internet connection, and making the medical records from patients who consented accessible to the research team. Still, internet disruptions continued throughout the course of the study, and some interviews had to be completed using hardcopies of the questionnaire, which were later entered and verified manually. This manual process took longer than the direct electronic data capturing. Data quality checks did not detect any differences in data entered manually versus collected electronically. At the end of the study, we provided certificates of appreciation to the partner clinics and interviewers.

### Outcome indicators: recruitment and participation

**Fig. 1:**
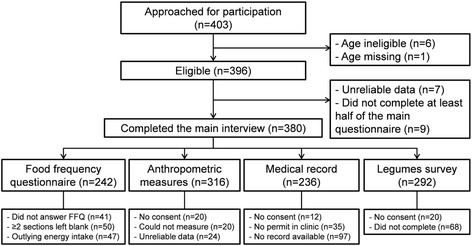
Recruitment and participation flowchart of the Puerto Rico Assessment for Diet, Lifestyle, and Diseases study

From July to November 2015, 403 participants approached our research assistants for participation in the study (Fig. 1). Of these, 396 met the inclusion criteria (7 were excluded due to ages younger than 30 or older than 75), and were invited to participate. Of these, 16 participants were excluded because the interviewer determined that the participant did not understand the questions or gave unreliable responses (*n* = 7) or for leaving more than half of the main questionnaire incomplete (*n* = 9). Thus, 380 participants (94% of those who approached the study) were included in the analysis for the main questionnaire.

The FFQ was started by 330 participants, or nearly 90% of the sample. However, the sample size with complete FFQ data was reduced to 252 after conducting standard exclusions for incomplete sections and outlying energy intake suggestive of misreporting. There were 316 participants with available anthropometric measures. Missing measures were due to not giving consent (*n* = 20), not being able to take the measure (*n* = 20; for example because the person had a physical limitation), or unreliable data (*n* = 24). Unreliable data for waist and hip measurements were more common among the first few interviews conducted by an interviewer.

While only 12 participants did not consent to having their medical record data abstracted, an additional 35 had signed documents at their respective clinic restricting any data sharing to third parties. Participants without medical records (*n* = 97) were visitors to the clinics or new patients. Thus, 236 had available medical record data. Finally, 292 completed at least half of the legumes questionnaire.

Most of the interviews were completed during the initial contact; 21 interviews were completed by phone, 16 in-person but at a later time (i.e., after the medical appointment or another day), and 16 could not be located after 3 attempts. Reasons included wrong phone numbers or disconnected lines. We restricted our final sample size to those with at least half of the main questionnaire complete. Incomplete questionnaires were mostly missing the psychosocial scales (*n* = 39), which was the last section. While 94% of participants initially consented to being re-contacted for future studies or additional question, 34 individuals requested later that they not be re-contacted; thus approximately 86% were willing to be re-contacted.

### Participants’ characteristics by site

More than half of participants were recruited from the community clinic (Health Pro Med), followed by the research clinic (FDI), and the city hospital clinic (Centro Multidisciplinario de Medicina del Hospital del Maestro) (Table 1). While the percent of participants with anthropometric and medical record data did not differ by clinic, there were significantly fewer participants with valid FFQ or with complete legumes questionnaire in the research clinic than the other two sites.

**Table 1:** Characteristics of participants of the Puerto Rico Assessment for Diet, Lifestyle, and Diseases, by site

Characteristic	Community clinic (*n* = 206)	Research clinic (*n* = 101)	City hospital clinic (*n* = 73)
*Recruitment indicators*			
Had valid food frequency data, %	73.8	35.6*	74.0
Had anthropometric measures, %	77.2	83.2	87.7
Had medical record data, %	53.9	65.3	64.4
Had legumes questionnaire, %	85.9	61.4*	72.6
Mean interview time, hour:minutes	1:25	1:37	1:52
Median interview time, hour:minutes	1:12	1:32	1:46
*Sociodemographics*			
Age, years	50.0 (11.7)	51.5 (10.3)*	55.5 (10.0)
Female, %	69.9	49.5*	75.3
Rural area of residence, %	3.9	32.7*	26.0
Ethnicity, %			
Puerto Rican	69.9	94.1*	97.3
Dominican	26.7	0	0
American/Other	3.4	5.9	2.7
Marital status, %			
Married/living with partner	44.3	36.1	47.8
Divorced/separated/widowed	20.7	20.6	21.7
Single	35.0	43.3	30.4
Education, %			
No schooling or <11^th^ grade	21.1	16.9*	0
12^th^ grade	26.1	29.5	8.5
Some college or higher	48.7	44.9	91.6
Household income, %			
$0–$10,000	70.9	68.0*	9.6
$10,001–$20,000	20.9	10.3	38.5
> $20,000	8.2	21.8	51.9
Employment, %			
Currently employed	35.4	26.7*	53.4
Retired/stay-at-home	51.5	48.5	38.4
Unemployed	13.1	43.1	10.3
Health insurance, %			
Public	63.4	49.5*	20.7
Private	27.3	46.3	72.4
No health insurance	9.3	4.2	6.9
Lives alone, %	20.9	37.6*	17.8
Receives food assistance^a^, %	67.0	49.0*	8.5
*Lifestyle and psychosocial factors*			
Abdominal obesity^b^, %	58.4	54.2*	77.3
High waist-to-hip ratio^b^, %	70.3	84.8*	83.3
Overweight/Obesity^b^, %	39.2	40.9	52.9
Sedentary physical activity^c^, %	40.2	42.9	54.1
Self-rated poor/fair dietary habits, %	28.3	38.6	26.0
Current smoker, %	18.2	28.1*	5.7
Current alcohol drinker, %	28.6	19.6*	31.4
Sleep, hours/day	6.7 (1.5)	7.2 (1.5)*	6.8 (1.5)
Perceived stress score^d^	22.5 (7.4)	20.4 (7.8)	21.0 (8.1)
Depressive symptoms score^d^	19.7 (12.6)	13.9 (10.9)*	16.3 (13.3)
Social support score^d^(Range 0–36)	23.4 (7.2)	26.4 (6.5)*	26.3 (7.0)
Diabetes emotional support score^d^(Range 0–40)^1^	13.7 (7.6)	15.2 (5.9)	14.1 (8.8)
*Self-reported medical diagnoses* ^*e*^			
Hypertension, %	37.8	36.5	47.1
Anxiety, %	28.0	31.9	31.5
Obesity, %	25.9	20.2*	43.9
Arthritis, %	24.4	23.7	30.1
Hypercholesterolemia, %	24.0	14.9*	35.2
Depression, %	20.9	21.5	26.0
Respiratory problems, %	23.3	18.5	16.9
Diabetes, %	17.2	22.2	28.8
Thyroid diseases, %	15.2	15.5	27.4
Gastrointestinal diseases, %	15.3	20.4	18.3
Pre-diabetes, %	14.6	15.3	16.7
Hypertriglyceridemia, %	12.1	14.1	22.9
Heart disease/stroke, %	10.2	13.2*	16.4

Shown as mean (standard deviation) or percent

^a^Determined as a member of the household currently receiving benefits from the Supplemental Nutrition Assistance Program

^b^Abdominal obesity defined as waist circumference >102 cm in men or >88 cm in women. High waist-to-hip ratio defined as ≥0.90 in men or ≥0.85 in women. Overweight/obesity defined as self-reported BMI ≥25.0 kg/m^2^

^c^Sedentary physical activity defined as a physical activity score < 30. Physical activity was captured using a modified Paffenbarger questionnaire; the score was calculated as the sum of hours spent on typical 24-h activities (heavy, moderate, light, or sedentary activity, and sleeping) multiplied by weighing factors that parallel the rate of oxygen consumption associated with each activity

^d^Perceived stress scale ranges from 0 to 56 with higher scores indicative of higher stress. Depressive symptomatology was assessed with the Center for Epidemiology Studies Depression (CESD) Scale which ranges from 0 to 60 with higher scores corresponding to stronger depressive symptomology. Social support was measured with the 12-item Interpersonal Support Evaluation List-12, which ranges from 0 to 36; greater scores relate to higher social support. The diabetes emotional support scale was only asked to participants with diabetes (*n* = 78) using the Diabetes Social Support Questionnaire-Family Version; the scale ranges from 0 to 25 and a higher score indicates higher diabetes support

^e^Self-report that the condition had ever been diagnosed by a doctor or health care provider

*p < 0.05 between clinics

Participants recruited at the community clinic were younger, had lower educational attainment and household income, and were more likely to reside in urban areas, have public or no health insurance, and receive government food assistance. Participants of Dominican ethnicity were recruited only at the community clinic. Individuals recruited at the city hospital clinic were older, more likely to be female, and had higher educational attainment, household income, current employment, and private health insurance. Finally, those from the research center were more likely to be male, reside in rural areas, be unemployed, and live alone.

Lifestyle and psychosocial characteristics varied by clinic, with participants with abdominal obesity more likely to be recruited from the city hospital, while participants with high waist-to-hip ratio were more often recruited at the city hospital and the research center. There were no significant differences in percent of participants with overweight/obesity status (based on self-reported measures), sedentary physical activity level, or self-reported poor/fair diet, by clinic; however these were relatively high for all sites. Participants from the research center were more likely to currently smoke but less likely to currently drink alcohol, and reported more hours of sleep. Participants from the community clinic had higher perceived stress and depressive symptomatology scores, and lower social support score. There were no statistically significant differences by clinic in prevalence of self-reported medical diagnoses except for obesity and hypercholesterolemia, which were higher among participants from the city hospital. Medical conditions were highly prevalent, with eight conditions reported by at least 20% of participants.

## Discussion

We document the challenges and opportunities that exist for conducting an observational study of chronic disease and lifestyle factors in PR, and we provide recommendations for similar efforts in the future (Table 2). First, the collaborative team must reasonably account for the time and effort needed to successfully implement a study of this nature. Preparing for PRADLAD took around one year; obtaining multiple IRB approvals took 4 months. The new policy from the National Institutes of Health on the use of a Single Institutional Review Board for multisite research may help ease this burden in subsequent projects [58]. The use of existing standardized protocols and questionnaires already validated and translated into Spanish saved time and effort. Only a few instruments had to be developed or adapted, and the goal is to validate them within PRADLAD for future use. It is important to ensure that the instruments utilized in a study are adapted and valid to the research setting, language, time, and context [59].

**Table 2:** Summary of challenges, opportunities, and recommendations for conducting collaborative epidemiological studies in Puerto Rico

*Challenges:*
• Multiple IRB approvals • Time-, cost-, and labor-intensive training and retraining of interviewers • Turnover of interviewers caused uneven recruitment flow and loss of potential interviews • Perception of long interviews from participants • Internet connectivity lost occasionally • Emotional reactions from some participants during sensitive questions • Re-contacting participants was not always possible • Errors in data entry and data quality checks • Extensive process of accessing and abstracting data from medical records • Specific characteristics of the source population of each site may bias the results
*Opportunities:*
• Motivation and engagement from partners and interviewers • Community clinics were easily accessible to participants and had necessary resources • Ample and positive interest and cooperation from participants, with high completion rates • Fast recruitment that met target goals in the expected time period • Standardized methods and questionnaires available (translated and validated in Spanish) • Extensive data across numerous topics collected in one interview • Real-time data capturing electronic system • General trust from participants in the research study and in culturally-sensitive interviewers • Incentives were appealing; snacks provided were received favorably • No adverse events reported • Recurrent process evaluation helped correct issues during the study • Exhaustive data quality checks corrected any errors and allowed for a clean dataset • Study established trust, feasibility, training, capacity-building, resources, and expertise • Multisite recruitment helped increase representation of the population
*Recommendations:*
• Work in partnership, and consider a multisite approach to increase representation • All collaborators prepare together written agreements at the beginning of the study • Leverage existing resources (such as medical records, interview rooms, internet connection) • Employ well-paid full-time interviewers to support steady recruitment and interview schedule • Assign site liaisons and senior coordinator to run logistics, administrative tasks, quality checks • Consider ways to shorten or expedite the interviews and procedures • Train/retrain culturally-sensitive interviewers frequently; train on sensitivity and friendliness • Include a pilot period to correct any on-field issues even if questions were pre-piloted • Schedule weekly or biweekly team meetings for process evaluation • Record feedback from interviewers and participants, qualitatively and qualitatively • Conduct frequent checks on recruitment logs and data quality • Incentive should match scope and effort of participation; these should be clearly conveyed • Provide additional incentives; i.e.: food, health literature, transportation, or giveaways • Use electronic data capturing, with paper-based questionnaires as back-up for Internet losses • Request multiple re-contacting information, and keep updated during the course of the study • Follow up with participants immediately if they need to complete the interviews later on • Provide certificates of appreciation or other recognitions to interviewers and partners • Report results to clinics and the community

While we screened fewer than the targeted 450 participants, the thorough training of interviewers and repeated data quality checks allowed us to minimize loss of observations and thus to keep nearly 94% of participants as our final sample size, more than the anticipated 360 participants after a 20% loss. The fairly fast fulfillment of recruitment goals suggests that our locations, scope of the study, and incentives were sufficiently convenient and attractive to volunteers. Strategies that have been shown to help increase participation and retention among minority groups include promptly providing incentives, having a familiar, accessible, and comfortable location, and developing tools to clearly explain the study’s purpose, importance, and procedures [60, 61].

Except for a few participants who answered the questionnaires hastily and requested not to be contacted again, participants had a positive attitude and they trusted in and cooperated with the study, which likely helped increase completion rates. Trust and cooperation were evidenced by the high percent of participants who consented to having their medical record accessed, as well as to being contacted for future studies or additional questions. The BPRHS reported a retention rate of 84% at 2-years follow-up [62], and a study of older adults (≥60y) in PR had 90% retention for the second wave [24], suggesting participants’ commitment to the studies. Other studies have suggested that Latinos have a strong willingness and trust to participate in biomedical studies, and are motivated by the perceived importance of the study topic and a desire to acquire and contribute to new knowledge [61, 63–65]. While material incentives are not always a strong motivation for participation in health programs among Puerto Ricans [66], they should be fair and commensurate to the time and effort of the study [60]. Additional incentives such as health literature or services, transportation, raffles, food, or pastime activities, may also be valuable [61, 66].

Fluctuation in recruitment during the course of a study is normal, but keeping weekly records of enrollment helped us identify an atypical drop and promptly rectify it by hiring more interviewers to keep an even recruitment flow. This, however, required new training sessions that, in addition to the re-trainings needed to reinforce protocols, became time- and labor-intensive and entailed extra cost and delay in recruitment. Nonetheless, interviewers were highly engaged with the study and with participants. PRADLAD trained 22 students in health-related fields about various epidemiological protocols, increasing their professional advancement and the prospect of being hired for similar projects. Recruiting experienced interviewers, and providing higher remuneration, may help increase productivity and retention of interviewers [67]. Moreover, our experience expands on that of community-based participatory research studies that suggest that minority students serving as research assistants are seen as trustworthy role models [68]. Interviewers of the same heritage or cultural values (i.e.: respect, personal approach, affability, same language) can further increase trust and participants’ satisfaction [61]. In fact, the emotional reactions from some participants when asked sensitive questions, may have been due to connecting with and trusting the interviewer. Showing respect for a patient is associated with the patient divulging feelings and personal information [69]. Training interviewers to sensitively handle personal questions is important for this population.

Process evaluation strategies helped us correct issues through the course of the study. This included having a pilot period to identify issues in the field, frequent debriefing team meetings, repeated data quality checks, and qualitative feedback from participants and interviewers. Requesting participants’ feedback was also done and recommended in the HCHS/SOL [61]. The questionnaire was shortened to decrease the length of the interview, but further efforts to keep interviews short and running swiftly, as well as including short breaks during the procedures, could be beneficial [61]. While participants were informed beforehand that the interviews would last 1.5 h, and we averaged 1:33 h after shortening it, participants still found the interviews long. Interviews were sometimes interrupted by loss of internet connection, which likely increased the perception of a long interview, as well as contributing to incomplete interviews. Fortunately, interviewers had hardcopies of the questionnaire and were able to continue, even if completing these took longer than the fast electronic data capture. Future studies should take advantage of real-time electronic data capture when feasible, as web-based technologies can facilitate, streamline, and lower the cost of data collection [70]. Having hardcopies may avoid losing or rescheduling participants. Finally, re-contacting participants was not always possible due to wrong phone numbers or disconnected lines. The BPRHS successfully located individuals because, in addition to the participant’s contact information, they recorded the information of two close contacts who could locate them, and they sent letters requesting them to call the study [29].

Thorough data quality checks helped us correct measurement and data entry errors, which also boosted quantity and quality of the observations. Although we had to delete some observations for low data quality, the number did not exceed those reported in other studies. For minority populations, who provide the same quality of information as other groups, population- appropriate data collection methods are particularly important to help maximize high-quality results [71]. Moreover, it has been posited that the quality of data reflects the field efforts expended [71], which were exhaustive in our study.

Cooperation, resources, and engagement from the partner clinics were substantial. Their staff helped advertise the study among patients and visitors, and provided access to the necessary resources. Still, retrieving medical record data took effort and time, and not all records had complete or accurate data, which was observed in another study in PR [72]. Use of electronic medical records facilitated the process. Working with multiple partners also helped us become better informed about the inherent characteristics of their source population and to contextualize results within the respective setting. This is important because survey research on minority populations benefits from a deep understanding of the sociocultural characteristics of the community or target group [71]. Two actions that reinforced the strong ties between the team were providing certificates of appreciation to the clinics and interviewers, and preparing a report to share with each clinic’s staff and patients. Further strategies to foster collaboration include having a written agreement of roles and responsibilities beforehand, and having designated liaisons at each site as well as a senior coordinator for the project.

Despite having standardized protocols, we observed differences in recruitment and main outcomes by clinic. Most participants were recruited at the community clinic; high participation is common in community settings [60]. Participants from the research center were less likely to complete the additional questionnaires, deserving further probing into possible reasons. We observed differential sociodemographic characteristics by site, likely due to the inherent source population of each clinic. For example, most of the women were recruited from the city hospital, which was originally designed to serve teachers (who are predominantly women), while the community clinic is located within a largely Dominican neighborhood. Having multiple sites with diverse backgrounds likely increased the representation of our participants and diminished biased results. A study of Puerto Ricans in Connecticut had a similar multisite recruitment approach in order to increases sociodemographic representation [73].

Importantly, chronic conditions and unhealthy risk factors were prevalent across all sites. This concurs with recent island-wide reports that show high prevalence of arthritis, depression, diabetes, overweight or obesity, and unhealthy lifestyle behaviors [1, 2]. Most of our sample had low income, high unemployment, and high educational attainment, as reported for the general PR population [1]. Our results also agree with reports from a recent cross-sectional study of similar design [27, 74]. This suggests that PRADLAD achieved wide representation, although the non-random sample design may limit generalizability of results.

## Conclusions

PRADLAD, a collaborative multisite cross-sectional study, helped establish trust, feasibility, training, capacity-building, resources, and expertise for conducting observational studies in Puerto Rico. While the cross-sectional convenience-sample design limits temporality and generalizability, the study accumulated data across multiple themes that will help answer gaps in knowledge of the relationship between diet, lifestyle, and diseases in PR, as well as generate hypotheses and preliminary evidence to design future studies and programs [7]. Working in partnership proved valuable to the study’s success. This type of collaborative work is essential to progress in public health initiatives, even in the face of challenges [4].

The documented high prevalence of unhealthy lifestyle behaviors and multiple chronic conditions warrants further research and intervention in PR. There have been calls to conduct large-scale epidemiological studies on risk factors, clinical care, and public health initiatives to prevent heart disease and related chronic conditions in PR [72]. This report has documented some of the operational challenges and opportunities that could help answer this call. Past epidemiological studies on similar topics have yielded important results. PRADLAD rigorously collected valid data on multi-level contributors to chronic diseases that will extend upon these findings to help improve the population’s health.

## Abbreviations

BPRHS: Boston Puerto Rican Health Study
CESD: Center for Epidemiological Studies – Depression
FDI: Fundación de Investigación de Puerto Rico
FFQ: Food frequency questionnaire
HCHS/SOL: Hispanic Community Health Study/Study on Latinos
IRB: Institutional Review Board
ISEL-12: Interpersonal Support Evaluation List-12
NHANES: National Health and Nutrition Examination Survey
PR: Puerto Rico
PRADLAD: Puerto Rico Assessment of Diet, Lifestyle, and Diseases
REDCap: Research Electronic Data Capturing
US: United States.
